# Risk factors and risk level assessment: Forty thousand emergencies over the past decade in China

**DOI:** 10.4102/jamba.v12i1.916

**Published:** 2020-11-09

**Authors:** Ning Ma, Yijun Liu

**Affiliations:** 1 Institute of Policy and Management, Chinese Academy of Sciences, Beijing, China; 2 University of Chinese Academy of Science, Beijing, China

**Keywords:** emergency, risk factor, risk matrix, risk level assessment, China

## Abstract

During a public emergency, which possibly evolves into a major public crisis, it is critical to quickly identify the main risk factors and assess the levels of risk, in order to efficiently manage the risks. In this study, about 40 000 emergencies in China over the past decade are investigated. Then, the five different types of risk factors are identified of these emergencies using the 5W1H methodology, including risk time (When), risk location (Where), risk population (Who), risk psychology (Why) and risk element (What), which lead to a risk matrix that is suitable for China’s national conditions. Based on this risk matrix, combined with expert knowledge, the Borda count and the analytic hierarchy process analysis, risk levels can be precisely assessed, solving ‘how to provide a solution’ (How), which provides decision-making guidance and facilitates prompt risk responses.

## Introduction

With the substantial influence and infiltration of the Internet, the online conflicts arising after major public emergencies are becoming increasingly intensified. Because the occurrence of emergencies and the transmission of risks are affected by many complicated factors, one of the key issues in emergency response is to conduct, based on historical emergency data, a systematic analysis and investigation on the risk levels of historical emergencies and to acquire trend patterns in data and text information, thus identifying various risk factors of emergencies. In this study, various risk factors are identified based on 44 274 public emergencies in China over the past decade, which form a ‘risk matrix’ suitable for specific national conditions, providing a foundation for further risk level assessment.

The 5W1H (what, when, where, who, why and how) analysis, also called the six Ws, is a complete methodology of problem solving, which was originally proposed by the American political scientist Lasswell in 1932. Through continuous use and improvement in later years, the 5WIH method was developed. The application of the 5W1H methodology to the study of scientific problems, that is, analysing from the six aspects of reason (Why), object (What), location (Where), time (When), person (Who) and method (How), enables researchers to think systematically and scientifically. The 5WIH method should be divided into two levels: ‘5W’ is the first level, corresponding to the fore-analysis, and ‘1H’ is the second level, corresponding to the back-end solutions. In the field of research on risk, the 5W1H methodology has been applied in the risk management of ancient buildings and metadata structure, and metadata is proposed for the risk management of architectural heritage using the 5W1H model in a context-aware application design (Lee et al. 2019). In the personalised safety instruction system for a construction site, the collected information is classified by the 5W1H method and transferred to particular workers according to their different characters (Tang et al. 2019). In the credibility evaluation of fake news, the 5W1H method is used to mutually evaluate each other based on the facts’ consistency (Ishida & Kuraya 2018). However, up until now there is a lack of analyses of the risks of public emergencies using the 5W1H methodology.

This article aims to identify the risk of emergencies and evaluate the risk level using the 5W1H framework. Specifically, this study has two research aims. The first aim is to label and analyse various risk factors of public emergencies, including risk time (When), risk location (Where), risk population (Who), risk psychology (Why) and risk element (What), based on the contents and characteristics of the online public opinions of a particular public emergency, combined with statistical analysis, text analysis and expert opinions. The second aim is to quantitatively assess the risk level of emergencies, in order to provide decision support for future risk responses, that is solving ‘how to provide a solution (How)’, based on the already identified risk factors, an improved risk matrix, the Borda count and the analytic hierarchy process (AHP).

## Literature review

The occurrence of major emergencies will lead to a number of casualties and property losses, as well as negative social impacts. Therefore, research on emergencies such as earthquakes (Zhang, Weng & Huang 2018), stampedes (Illiyas et al. 2013) and terrorist attacks (Liu 2018) has increased, which mainly focuses on the following three aspects.

The first aspect is risk decision analysis of emergencies. After major emergencies took place, emergency managers or decision-makers should make correct decisions within a short time, with a view to reducing subsequent negative impacts. First, it is necessary to identify risks (Qing, Huimin & Yanling 2012). Then aiming at the purpose of optimising decisions, multiple attribute utility theory (Hämäläinen, Lindstedt & Sinkko 2000), risk decision method based on data mining of public attribute preferences (Xu, Yin & Chen 2019), risk decision analysis method based on cumulative prospect theory (Liu, Fan & Zhang 2014), group analytic network process approach (Levy & Taji 2007), fuzzy optimisation method for multi-criteria decision-making (Fu 2008) and decision-making method based on distance are proposed (Yu & Lai 2011).

The second aspect is risk level assessment of emergencies. Risk is the forerunner of crisis, and crisis is developed risk. A graded assessment of the risk level of emergency is a prerequisite for preventing further crises. The perspective of risk level assessment research has mainly focused on areas including the environment, the chemical industry and food safety. There are very few studies focusing on the risk level assessment of social emergencies, amongst which the research on terrorist attacks and violent mass incidents focuses on psychological trauma recovery (Gibert et al. 2015). To assess the risk level, a risk matrix is commonly used for a comprehensive assessment of risk through the probability of risk occurrence and the severity of a hazard. Because of the feasible way to express risk and the easy-to-use feature, a risk matrix is a widely applied tool for semi-quantitative risk assessment (Ni, Chen & Chen 2010). At present, the risk matrix method is mainly used for risk assessment in the fields of safety accidents (Skorupski 2016) and engineering project construction (Duan et al. 2016). Amongst them, some studies combine the fishbone diagram (Luo, Wu & Duan 2018), fuzzy AHP (Hsu, Huang & Tseng 2016) or Technique for Order of Preference by Similarity to Ideal Solution (TOPSIS) approach (Yazdi 2018) to construct the risk matrix. However, no studies have yet used the risk matrix method for the risk assessment of serious emergencies.

The third aspect is the public opinion risk of emergencies. With the development of Internet technology, more and more people are accustomed to using the Internet to search for risk information (Jin, Liu & Austin 2014). In this case, after the occurrence of major emergencies, on the one hand, online media quickly spread relevant information to satisfy the public’s need for information (Bunz 2010) and improve their risk perception ability (Hong, Kim & Xiong 2019); on the other hand, online media also become main communication media of rumours, which leads to secondary public opinion risks (Huo, Huang & Fang 2011). After a major emergency, rumours or wrong information generated and propagated during communications amongst the public may easily cause panic or social instability. Hence, to well manage emergencies and risks, it is also essential to make full use of the role of the Internet (Lachlan et al. 2016; Panagiotopoulos et al. 2016).

Although the overall risk level during a crisis is relatively high, prevention and control during the early stage of public communication during a crisis may avoid the risk of public opinion escalating the crisis to the greatest extent. Different from the above research on a certain type of emergencies or a certain emergency, this research is supported by a large amount of historical data. Based on historical data of 44 274 public emergencies in China over the past decade, risk factors are identified and evaluated to quantitatively label various risk factors, in order to build a risk matrix that is suitable for China’s national conditions. This matrix is then used to predict and evaluate the risk level of new incidents, from a perspective of controlling risk from the source.

## Data collection and research methodology

### Data collection and processing

Using computer-based data acquisition supplemented with manual screening, we regularly monitor several major domestic news websites in China and count the major public emergencies in various regions throughout Chinese mainland. The collected data include two dimensions: the ‘event dimension’, which refers to information about the name, type, time and place of a particular incident, and the ‘public opinion dimension’, which contains information about the online posts, person who posted, the release time, and how many people have read, commented or given ‘like’ to a particular post (Table 1). For the ‘event dimension’, the time frame for data collection is from 2009 to 2018, with a total collection of 44 274 public emergencies. For the ‘public opinion dimension’, 50 major emergencies are selected from the above incidents for analysis. Table 2 lists the major emergencies that occurred in China over the past decade.

**TABLE 1:** The collected data of a specific case (example).

Title	Dimension	Item	Text or data	Supporting materials
Children stabbed to death in a primary school	Event dimension	Event type	Public safety	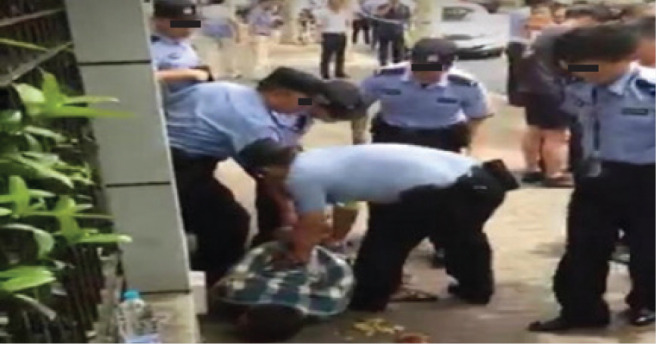
		Occurrence time	06/28/2018	
		Occurrence location	Shanghai	
		Number of deaths	2	
		Number of injured	2	
		Economic loss	-	
	Public opinion dimension	Communication platform	Micro blog	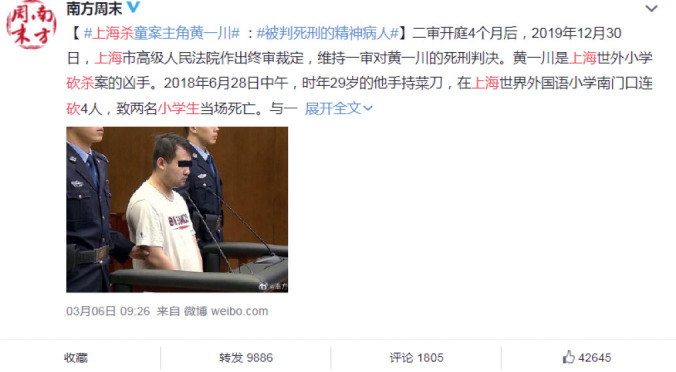
		Release time	03/06/2020	
		Number of forwarded messages	9886	
		Number of reviews	1805	
		Number of likes	42 645	
		Views of netizens	-	Killing people pay for their lives; Guardians should also be punished; Mental illness is not an excuse

**TABLE 2:** List of 25 major emergencies over the past decade in China.

Time	Title	Type	Location
06/05/2009	Bus arson in Chengdu	Public safety emergency	Sichuan
06/17/2009	Group violent incident in Shishou	Public safety emergency	Hubei
03/23/2010	Campus murder in Nanping	Public safety emergency	Fujian
04/14/2010	Yushu earthquake	Natural disaster	Tibet
11/15/2010	Shanghai high-rise residential fire incident	Accident calamity	Shanghai
03/25/2011	Henan’s ‘lean’ event	Public health emergency	Henan
07/21/2012	Torrential rain in Beijing	Natural disaster	Beijing
05/03/2013	A girl’s death at Jingwen mall in Beijing	Public safety emergency	Beijing
06/07/2013	Bus arson in Xiamen	Public safety emergency	Fujian
11/22/2013	Explosion of oil pipe line in Qingdao	Accident calamity	Shandong
03/01/2014	Terrorist attack at railway station of Kunming	Public safety emergency	Yunnan
07/05/2014	Bus arson in Hangzhou	Public safety emergency	Zhejiang
08/02/2014	Explosion at a chemical plant in Kunshan	Accident calamity	Zhejiang
12/31/2014	Stampede in Shanghai Bund	Accident calamity	Shanghai
08/12/2015	Explosion in Binhai New Area of Tianjin	Accident calamity	Tianjin
03/19/2016	The illegal vaccine in Shandong	Public health emergency	Shandong
08/08/2017	Jiuzhaigou earthquake in Sichuan	Natural disaster	Sichuan
06/22/2017	Hangzhou babysitter arson	Public safety emergency	Zhejiang
11/22/2017	RYB kindergarten child abuse incident	Public safety emergency	Beijing
11/28/2017	Tuberculosis epidemic in a middle school	Public health emergency	Hunan
04/27/2018	Students killed in Mizhi County, Yulin City	Public safety emergency	Shaanxi
06/20/2018	Girl jumped off building in Qingyang City	Public safety emergency	Gansu
06/28/2018	Children stabbed to death in a primary school	Public safety emergency	Shanghai
10/28/2018	Chongqing bus crashed into a river	Accident calamity	Chongqing
07/15/2018	Changchun Changsheng biological vaccine	Public health emergency	Jilin

RYB, red yellow and blue.

### Risk factor identification method

**FIGURE 1:**
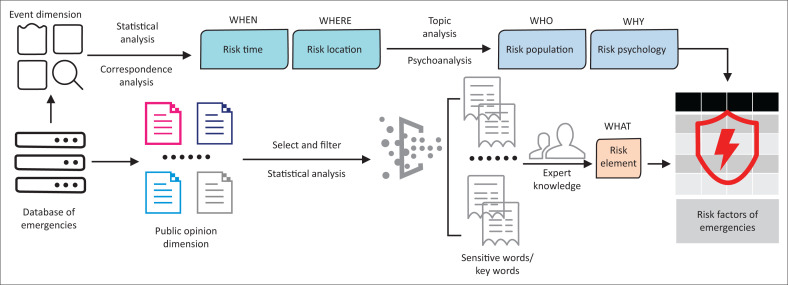
Method of labelling risk factors of emergencies.

To identify the risk factors of an emergent event, first the location and time of the event are identified through statistical analysis and correspondence analysis (CA) (Benzécri 1973; Hirschfeld 1935) of the ‘event dimension’ data. Second, through analysing the main content of the emergency, relevant population at risk and their psychological drives can be identified. Then, in terms of risk element identification, the text mining of the public opinion information from each type of emergency is conducted through statistical analysis and expert knowledge. Specifically, the keywords related to risk elements for emergencies are identified and analysed, and then, combined with expert knowledge, these risk elements are screened and graded. The relationship between different risk elements can be analysed by complex network. By combining these analyses, key risk factors can be identified of emergencies (Figure 1).

The main methods involved are as follows:

- Correspondence analysis: CA is a method of data visualisation that is an exploratory multivariate technique that converts a data matrix into a particular type of graphical display in which the rows and columns are depicted as points (Greenacre & Hastie 1987). The ‘occurrence time – emergency’ and the ‘occurrence site – emergency’ could be calculated using this method.
- Text mining: Text mining is a technique for extracting meaningful information from data in text form. This technique can find new information in human character based data by extracting context and meaning using natural language and document processing techniques (Bunescu & Mooney 2007). For example, the risk elements (keywords) in public opinion information can be found by word segmentation, part-of-speech tagging, new word detection, etc.
- Complex network analysis: A complex network is a network that represents high complexity, and its development is attributed to the development of applied mathematics, including graph theory and topology. A concrete complex network can be abstracted as a graph composed of a node set and an edge set. Statistical characteristic analysis of a complex network can interpret many complicated phenomena in life (Watts & Strogatz 1998). In this study, ‘node’ represents the psychological type, whilst ‘edge’ represents the correspondence relationship.

Remark: In this study, ‘risk factors’ correspond to the ‘5W’ of the 5W1H method and ‘risk element’ just represents the ‘WHAT’ (1W) of the 5W1H method, so ‘risk factors’ contain ‘risk element’.

### Risk level assessment method

**FIGURE 2:**
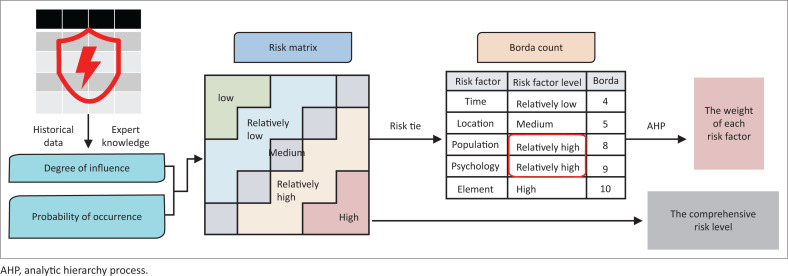
Method of evaluating the risk level of emergencies.

Based on the identified risk factors of various emergencies, combined with expert opinions, a risk matrix is constructed. However, when applying the risk matrix for risk assessment, different risk factors may appear at the same risk level, resulting in a ‘risk tie’. To address this issue, the Borda count is introduced to rank different risk factors according to their importance. Because the value of the Borda count itself is a relative value, generating a pairwise comparison based on the importance of different risk factors, a two-dimensional judgment matrix can be formed, which provides a quantitative basis for further determination of the weight of each risk factor using AHP (Figure 2).

The main methods involved are as follows:

- **Risk matrix:** Risk matrix is a structured approach to identify the importance of risk in project management. It assessed the potential impact of risk through a simple operation method combined qualitative and quantitative analysis. Traditional risk matrix level is determined by a combination of risk probability of occurrence and severity of the consequences.
- **Borda count:** Borda count is a well-known social choice method frequently used for group decision-making problems (Zarghami 2011); this method can determine the winner of an election by giving each candidate a certain number of points corresponding to the position in which he or she is ranked by each voter. In this study, the Borda count is applied to rank risk factors to ensure the different risk factors each had a unique number.
- **Analytic hierarchy process:** AHP is a multiple-criteria decision-making tool that has been used in almost all the applications related with decision-making (Saaty 1987). Analytic hierarchy process is an Eigenvalue approach to the pair-wise comparisons (Vaidya & Kumar 2006). In this study, AHP will be applied to determine the weight of each risk factor.

## Risk factors identification of emergency

### Types of risk factors

Taking the initial cause of an emergency as the starting point and guided by the ‘5W1H’ method, we analysed the risk factors of an emergency based on its innate features and related public opinions. Then, we evaluate the impact of different risk factors on the development of the emergency, labelling the risk factors by type.

- **Risk time (When):** According to observation, the emergencies show significant non-equilibrium across time variables and a centrality of times of high occurrence. In addition, emergencies that occur during risk times or sensitive times are more likely to cause public concern and exert a substantial social impact, including the dates of major historic events, of important national conferences or events, and of important holidays and festivals.
- **Risk location (Where):** Historical data show that the types and occurrences of emergencies vary from region to region and that high-risk areas of emergencies are risk locations. For example, ethnic areas at the national border are geographically remote. Their natural environment is harsh. In addition, their ethnic, religious and cultural relationships are complex. The eastern part of China exhibits a higher economic development level, higher cognitive capabilities of the locals, and better Internet development technologies than do ethnic areas. Both of the above-mentioned areas have become high-risk areas within the nation.
- **Risk population (Who):** The type of stake-holding populations in emergencies exerts a crucial effect on the evolution of public opinion. If an emergency involves a ‘labelled group’, certain emergencies may quickly stir up waves in the public opinion field. For example, in some netizens’ minds, ‘civil servants’ equals corruption, ‘police’ equals illegality and involvement with gangs, ‘urban management officials’ equals violent law enforcement, and ‘the children of the powerful and wealthy’ equals arrogance.
- **Risk psychology (Why):** In the process of the development of online public opinion regarding emergencies, public psychology is often the internal momentum for public opinion evolution. Common risk psychology includes bystander effect, victim mentality, anxiety and fear psychology, and habitual suspicion psychology. For example, habitual suspicion would create mistrust between the public and the government, making the public doubt everything the government does and spread rumours.
- **Risk element (What):** When an emergency occurs, the involved intrinsic sensitive element can resonate with the online public opinions, thus deriving new risk elements. For example, ‘corruption’ might be derived from an incident involving ‘the death of an official from a fall’ whilst ‘rape’ might be derived from an incident involving ‘the death of a girl from a fall’. Such assumptions may increase the destructive power of the event.

### Risk location and risk time

*The location–event analysis* and *the time–event analysis* are constructed by counting the frequency of different types of emergencies in different spatial and time dimensions. Then, CA is carried out on the two dimensions, respectively, and the relationship is explored from the perspective of ‘location–time–event’ in order to grasp the location–time coupling of certain emergencies, thus labelling the high-risk locations and high-risk times.

**FIGURE 3:**
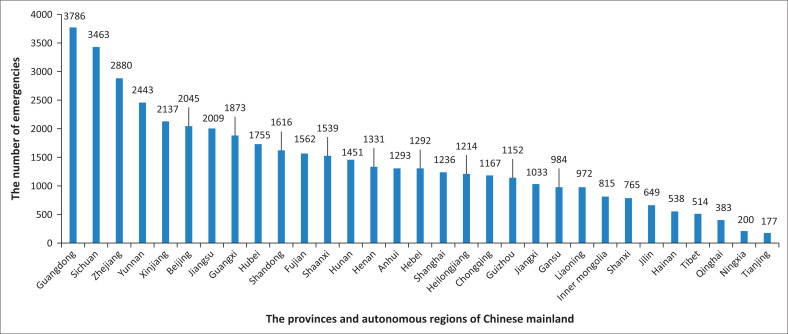
Statistics on the number of emergencies in various regions of Chinese mainland (2009–2018).

**FIGURE 4:**
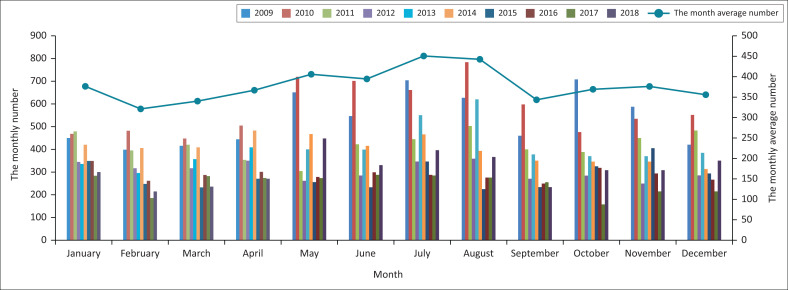
Statistics on the number of emergencies in each month in Chinese mainland (2009–2018).

First, high-risk locations and high-risk times of emergencies are analysed based on data collection and statistics (Figures 3 and 4). It can be seen from Figure 3 that Guangdong Province has the highest incidence of emergencies, where more than 3 500 emergencies occurred within 10 years. Sichuan Province and Zhejiang Province are also areas with high incidences of emergencies. More than 3000 emergencies occurred in Sichuan within 10 years, and more than 2500 occurred in Zhejiang within 10 years. For Yunnan, Xinjiang, Beijing and Jiangsu, more than 2000 emergencies occurred in each location within 10 years. It can be seen from Figure 4 that in the past decade, the months with the higher average number of emergencies per month are July, August, May and June. In general, summer is a period with a high incidence of emergencies.

**FIGURE 5:**
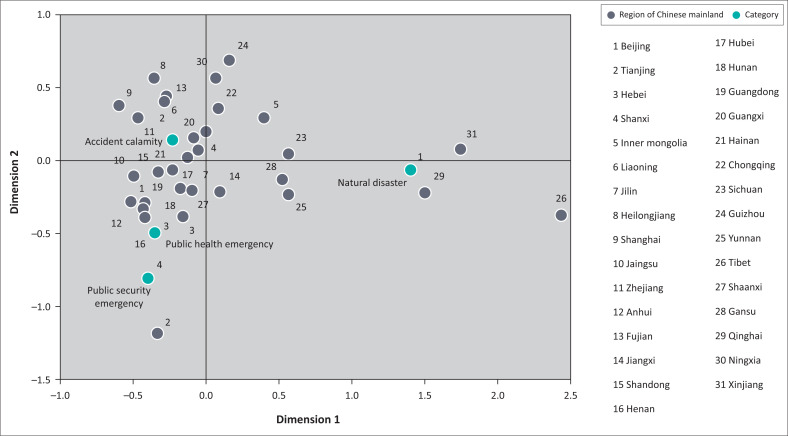
‘Location–event’ correspondence analysis of emergencies.

According to the type of emergency, the ‘location–event’ two-dimensional matrix and the ‘time–event’ two-dimensional matrix are constructed. With the aid of Statistical Package for Social Sciences (SPSS) software, the corresponding analysis is carried out to identify high-risk locations and high-risk times for different types of emergencies (Figure 5). Public emergency is consisted of natural disaster, accident calamity, public health emergency and public security emergency.

It can be seen from Figure 5 that the ‘natural disaster’ category has a high correlation with various regions in western China, such as Qinghai, Xinjiang, Yunnan, Sichuan and Tibet. Because of the relatively harsh natural conditions in these regions, these areas are vulnerable to natural disasters. The ‘accident calamity’ category bears no obvious regional characteristics. By comparison, the probability of an accidental disaster is high in resource production areas and industrial manufacturing areas. Thus, the geographical correlation is relatively high in places such as Shandong, Liaoning and Tianjin. ‘Public health’ incidents are highly correlated with Hebei, Anhui and Henan because serious ‘vaccine scandals’ occurred in these areas, drawing relatively high public concern. ‘Public security’ incidents are similar to public health incidents. Areas such as Tianjin, Hebei and Anhui have high correlations because of the high population concentration, as well as the prominent social contradictions brought about by uneven economic development.

**FIGURE 6:**
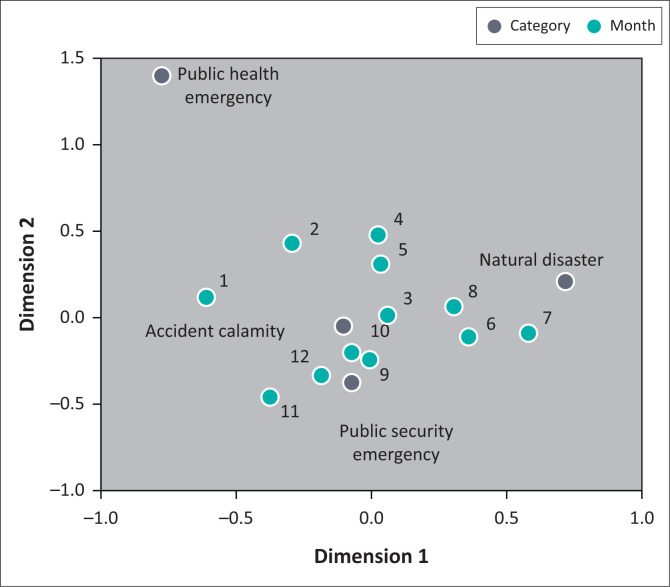
‘Time–event’ correspondence analysis of emergencies.

Figure 6 shows the temporal pattern of the probability of occurrence of different types of emergencies. ‘Natural disasters’ occur mostly during the summer months of June, July and August. This is closely related to the changing summer weather, creating extreme weather conditions. For example, natural disasters such as heavy rain, floods, typhoons, drought, landslides and debris flows are all closely related to the strong convective weather in summer. ‘Accident calamity’ occurs mostly in March, during the spring, and ‘public health’ incidents occur mostly during January and February when New Year and Spring Festival are celebrated. During these traditional Chinese festivals and holidays, people travel and visit relatives and friends, making traffic accidents more likely to occur. Large-scale celebrations or gatherings involving large numbers of participants are likely to bring about infectious diseases/epidemics or food safety problems. ‘Public security’ incidents occur mostly during the fall and winter months of September, October, November and December.

### Risk population and risk psychology

#### Risk population

**FIGURE 7:**
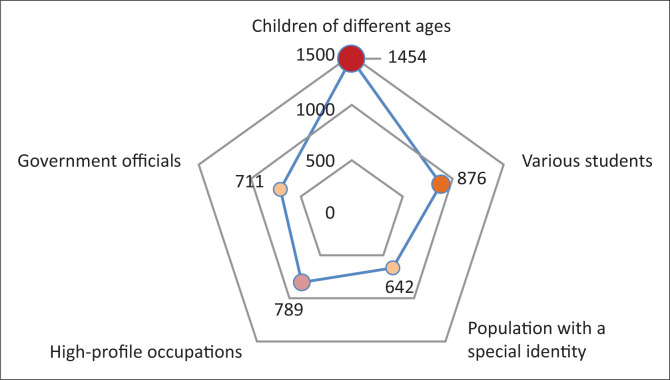
Different types of risk populations of emergencies.

Emergencies are more likely to cause public concern when they involve a certain kind of risk population. The ‘risk population’ may have stigma effects or sympathetic effects. According to the statistics for different groups of people involved in emergencies in the past decade, risk populations can be grouped into the following five categories (Figure 7): government officials (governor, mayor, county magistrate, etc.), high-profile occupations (police, doctor, city inspector, etc.), population with a special identity (adoptive mother, adoptive father, the rich second generation, etc.), children of different ages (infant, baby, child, etc.), and various students (pupil, middle school student, college student, etc.). According to the types of occupation, government officials and high-profile occupations are selected. According to the social label, some sensitive groups are selected to form the population with a special identity. According to the age, children and various students are selected. The first three are often stigmatised whilst the latter two are often sympathised.

**FIGURE 8:**
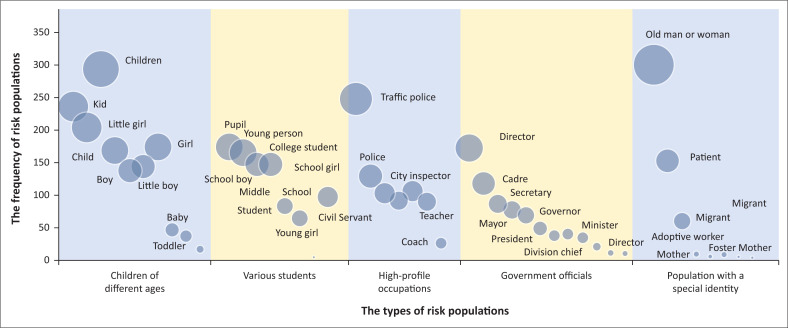
Frequency of various types of risk populations of emergencies.

Statistics on the frequency of the above-mentioned risk populations of event title have revealed the following facts. The ‘children of different ages’ appear the most (1454 occurrences) and draw the most concern, followed by the ‘various students’ population (876 occurrences). The majority of students are minors who are a vulnerable population that is more likely to attract attention. For example, this population includes primary school students, middle school students, schoolgirls, young girls and teenagers. Next are the ‘high-profile occupation’ population (789 occurrences) and the ‘government officials’ population (711 occurrences), primarily involving populations that are prone to conflicts between police and civilians, conflicts between the government and civilians, and conflicts between doctors and patients. For example, this population may include traffic police, urban management officials and doctors. Last is the population with a special identity (789 occurrences), referring mainly to a ‘labelled’ group of people. Examples include the elderly are extortionists by playing the role of a victim; patients are hard to deal with; migrant workers are uncultured and ill-mannered; and children of the powerful and wealthy are arrogant (Figure 8).

#### Risk psychology

During emergencies, different populations may have different interests, thus creating different risk drives, with different psychological types and psychological evolution processes. In the same emergent event, a variety of different types of risk psychology may be intertwined, causing the public opinion concerning the matter to worsen or escalate the emergency into a crisis. Through the analysis of the theory of group polarisation, the theory of the spiral of silence (Noelle-Neumann 1974), Maslow’s hierarchy of needs (Maslow 1943) and other relevant psychological theories, 10 types of risk psychology concerning the public opinion of major emergencies are summarised in Table 3.

**TABLE 3:** Ten types of psychology concerning public opinion of emergencies.

Type	Interpretation
Unbalance	In an emergency, some netizens compare their own situation with a certain standard or a reference point and determine that they are at a disadvantage. Thus, they feel they are deprived and label themselves as a member of ‘a vulnerable group’. Unbalance psychology is caused by a sense of being deprived.
Questioning	The questioning by netizens is mainly manifested in their ‘habitual suspicion’, ‘habitual opposition’, ‘habitual criticism’, etc. Under the influence of questioning, these netizens often start criticising and accusing officials’ behaviour without seeking the truth.
Primary effect	Generally, it is related to ‘the importance of first impressions’ or ‘preconceived ideas’, which means that in the social activities that facilitate the formation and dissemination of public opinion, the first impression you give to the other party has an important influence on your future relationship with her or him. In an emergency, the initial information that netizens receive largely determines their basic understanding and judgment of the event.
Profit-seeking	After an emergency, some media or individuals often deliberately hype the event just to attract other netizens’ attention, create conspicuous network traffic and make profits.
Conformity	Conformity is a common psychosocial phenomenon referring to the fact that an individual’s attitudes and behaviours are influenced by other netizens. These people tend to follow the majority opinion.
Onlooker	In the Internet age, the development of social media such as Weibo and WeChat has facilitated the participation of general netizens in the evolution of public opinion. These netizens do not publish comments with personal emotions regarding the event. They merely forward or like it, being a bystander following the onlooker psychology.
Resentment	Resentment is a negative social psychology. Currently, it is mainly manifested as resenting officials, the powerful and the wealthy. The resentment of officials reflects civilians’ distrust of government officials. Resentment of the powerful reflects the general public’s misinterpretation and misunderstanding of rights and privileges. Resentment of the rich reflects a negative reaction to the gap between the rich and the poor.
Venting	When people accumulate negative emotions or negative energy in their daily life and work, they often use network information about an emergency as a way of venting in order to obtain an emotional release.
Anxiety	Anxiety is a complex emotional state in which feelings of anxiety, restlessness, care and depression are intertwined toward something that is closely related to themselves and that is about to happen.
Curiosity	Curiosity is human nature. People tend to show curiosity in regard to things that are new and interesting. After an emergency, random browsing, searching for the truth and discussion of the event on the Internet are a manifestation of curiosity.

**FIGURE 9:**
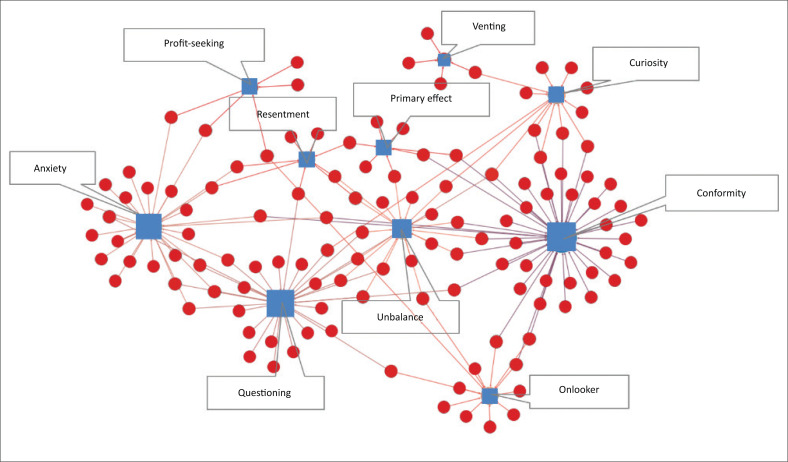
Network of psychological characteristics of emergencies.

We select typical cases of major emergencies that have occurred over the past decade and analyse the corresponding psychological types based on the comments of people. Then, we apply the social network analysis to draw a network diagram of the relationship between emergencies and psychology (Figure 9). Applying the complex network structure index to measure the relationships, the following is found: (1) The average degree of the nodes was 1.25. The psychological type of ‘Conformity’ is the highest and had a connection relationship with 42 emergencies. It is followed by the psychological types ‘Anxiety’ and ‘Questioning’; (2) The modularity index was 0.614, which was divided into eight modules according to the network structure. Basically, a module corresponds to a psychological type.

Combined with specific cases, there are three findings. First, when a particular incident involves governmental departments or public servants, the public often questions the incident and has psychological imbalance towards the investigation of the incident. The public habitually suspect government behaviours and always label the public as a ‘vulnerable group’ under the influence of their psychological imbalance. Second, in general, for major natural disasters or traffic accidents, the majority of the public manifests conformity and onlooker psychology. For example, in a major traffic accident, the public generally expresses mourning and regret for casualties caused by the accident. Third, the majority of the public manifests anxiety in emergencies related to the safety of their own lives. For example, for the ‘vaccine scandal’ and for ‘being murdered on a car ride booked online’, the public shows concern about their own safety.

### Risk element

When risk elements (or sensitive words) are involved during the dissemination of public opinion, civilians’ feelings are affected, their attentions drawn, and complaints and negative emotions generated. For example, the vaccine scandal event in March 2016 was published in *Pengpai News* (thepaper.cn) on 18 March with the title ‘Hundreds of millions of yuan’s worth of vaccines spread into 18 provinces without being refrigerated: This is murder, Shangdong issued a letter of investigation’. The word ‘murder’ triggered public attention, and public sentiment quickly spread. However, as early as 23 February, Xinhua News Agency published a news article titled ‘Jinan police cracked the case of illegally operating human vaccines worth 570 million yuan’, which did not stir up huge waves of public opinion.

**FIGURE 10:**
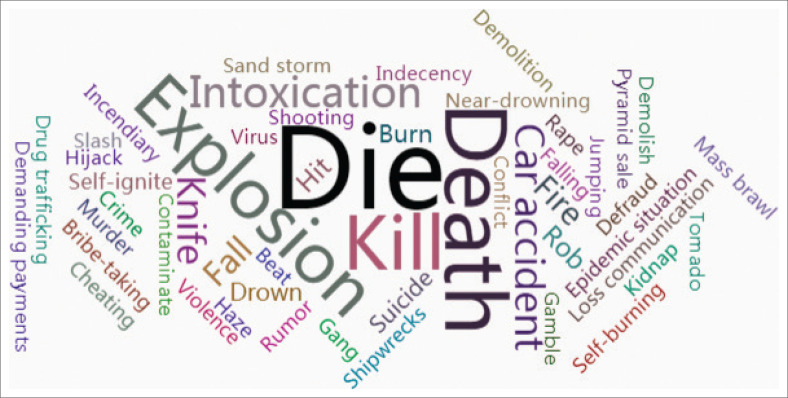
Risk elements for public opinion transmission in emergencies.

Through the text analysis of the communication content of public opinion regarding major emergencies in recent years, combined with expert knowledge, the risk elements that can easily cause the outbreak of public opinion are identified and shown in Figure 10. For example, there are words related to personal safety such as ‘Die’ (9015), ‘Death’ (1908), ‘Kill’ (939) and ‘Suicide’ (224). There are words that are likely to cause concern amongst vulnerable groups such as ‘Hijack’ (76), ‘Rape’ (56) and ‘Indecency’ (52). There are also words that may cause public panic such as ‘Intoxication’ (935), ‘Virus’ (54) and ‘Slash’ (37). The numbers indicate the frequency of the different risk elements in the title of emergencies.

## Risk level assessment of emergency

Based on the various types of risk factors identified from the historical emergencies, we first determine the degree of influence and probability of occurrence of different risk factors and construct a risk matrix to evaluate the level of risk factors. Then, we use the Borda count to sort and evaluate the importance of various risk factors, obtaining the sorting results of the importance of various risk factors. Finally, according to quantitative ranking results based on risk factor importance, we establish an AHP judgment matrix, determine the weight of each risk factor and ultimately obtain the comprehensive risk level.

### Evaluation of various risk factors to build the risk matrix

In the case of different risk levels and risk occurrence probability, the resulting risk matrix is different. In this article, the influence of different risk factors is divided into five levels, and the risk occurrence probability is also divided into five levels, forming a 5 × 5 risk matrix. Specifically, the five risk influence levels are ‘limited’, ‘slight’, ‘moderate’, ‘serious’ and ‘crucial’. The risk influence is divided into five levels mainly based on the occurrence frequency of different risk factors in the 40 000 emergencies over the past decade in China. For example, the highest frequency words are ‘Die’, ‘Death’ and ‘Kill’ about risk element, so these words belong to the ‘crucial’ level. The five levels of risk occurrence probability are ‘highly unlikely (0% – 10%)’, ‘unlikely (11% – 40%)’, ‘likely (41% – 60%)’, ‘very likely (61% – 90%)’ and ‘extremely likely (91% – 100%)’. Thus, a risk matrix for emergencies is created (Table 4). To further satisfy the decision-makers’ grasp and control of the risk in emergencies, this article increases the risk classification scale to five levels (three risk levels in the traditional risk matrix), namely ‘low’, ‘relatively low’, ‘medium’, ‘relatively high’ and ‘high’; in addition, the quantitative criteria are determined to be 0.1, 0.3, 0.5, 0.7 and 0.9, respectively.

**TABLE 4:** Risk matrix of emergencies.

Degree of influence (*I_i_*)	Risk factor (*R_i_*)					Probability of occurrence (*P_i_*)				
	Risk time	Risk location	Risk population	Risk psychology	Risk element	Highly unlikely	Unlikely	Likely	Very likely	Extremely likely
Limited	Non-high incidence period and sensitive period	Non- high incidence area	Not involving a common risk population	Not involving a common risk psychology	Not involving a common risk element	Low	Low	Relatively low	Relatively low	Medium
Slight	Other high-incidence period	Other high-incidence area	Ordinary public officer and civil servant	Onlooker, conformity, curiosity	Fraud, extortion, rumour, epidemic, etc.	Low	Relatively low	Relatively low	Medium	Relatively high
Moderate	High-incidence period June–August	Beijing, Shanghai, Tianjin, etc.	Teacher, student, child, etc.	Primary effect, imbalance, profit-seeking	Kidnapping, terrorist attack, self-immolation, etc.	Relatively low	Relatively low	medium	Relatively high	Relatively high
Serious	During major holiday and festival	Sichuan, Zhejiang, Yunnan, etc.	Urban management officer, doctor, migrant worker, etc.	Anxiety, venting	Cult, hacking, sexual harassment, lewdness, etc.	Relatively low	Medium	Relatively high	Relatively high	High
Crucial	During the same period when major historical events occur	Guangdong, Xinjiang, Tibet, etc.	officer, traffic police, girl, etc.	Resentment, questioning	Death, killing, explosion, shooting, etc.	Medium	Relatively high	Relatively high	High	High

To further illustrate the risk level assessment process, we selected a typical case to show details of the analysis. On 28 June 2018, near a primary school in Shanghai, an unemployed man enacted ‘revenge on society’ by slashing three boys and one female parent. Two of the three injured boys died. Based on the ‘risk matrix of emergencies’ (Table 4), experts in relevant fields are invited to evaluate the influence degree and probability of risk factors, resulting in the risk matrix shown in Table 5.

**TABLE 5:** Risk factor assessment results regarding ‘Shanghai slashing of primary school students’.

Risk factor (*R_i_*)	Degree of influence (*I_i_*)	Probability of occurrence (*P_i_*)	Risk factor level (*RL_i_*)	Risk factor level quantised value (*RL_i_*)
Risk time: June	Moderate	11% – 40%	Relatively low	0.3
Risk location: Shanghai	Moderate	41% – 60%	Medium	0.5
Risk population: Students	Moderate	61% – 90%	Relatively high	0.7
Risk psychology: Venting	Serious	61% – 90%	Relatively high	0.7
Risk factor: Slashing	Crucial	61% – 90%	High	0.9

### Quantitative ranking of the risk factors by the Borda count

For risk factors regarding the ‘Shanghai slashing of primary school students’ incident, both the ‘risk population’ and ‘risk psychology’ are ‘relatively high’, resulting in a ‘risk tie’, which means that the same risk level occurs for different risk factors. In this case, the Borda count is used to quantitatively rank the relative effect of the above five risk factors on the overall risk. The Borda count formula is as follows:

$$b_{i}=\sum_{k=1}^{2}\left(N-R_{ik}\right)$$ [Eqn 1]

$$b_{ri}=\sum_{j=1,j\neq i}^{4}M(b_{j}>b_{i})$$ [Eqn 2]

where *N* is the total number of risk factors to be evaluated, *K* is the evaluation criterion, *M* is the number of *k*, and in this article *M* = 2. *k* = 1 indicates the degree of influence of risk factor, *k* = 2 indicates the probability of occurrence of risk factor and *R_ik_* indicates the number of risk factors that are higher than the risk factor *i* in criterion *k. b_ri_* represents the Borda value of risk factor *i*, that is the number of other risk factors that are more important than this risk factor.

The above Borda count is used to evaluate the importance of each risk factor of a specific case. The results are shown in Table 6.

**TABLE 6:** Ranking of the importance of each risk factor in the ‘Shanghai slashing of primary school students’ incident (Borda value).

Risk factor (*R_i_*)	Degree of influence (*I_i_*)	Probability of occurrence (*P_i_*)	*R_ik_K* = degree of influence	*R_ik_K* = probability of occurrence	Borda value (*b_i_*)	Borda ordinal value (*b_ri_*)
Risk time	Moderate	11% – 40%	2	4	4	4
Risk location	Moderate	41% – 60%	2	3	5	3
Risk population	Moderate	61% – 90%	2	0	8	2
Risk psychology	Serious	61% – 90%	1	0	9	1
Risk element	Crucial	61% – 90%	0	0	10	0

According to the Borda count (*b_ri_*) (Equation 1 and 2), different risk factors in the above incident have different degrees of influence. The most important one is ‘risk element’, followed by ‘risk psychology’ and ‘risk population’. ‘Risk location’ and ‘risk time’ have the least impact.

### Determination of the comprehensive risk level by the analytic hierarchy process

As the Borda value itself is a relative value, it is easy to compare the importance of different risk factors amongst pairs and construct a two-dimensional judgement matrix (Equation 3). An AHP method is then applied to determine the weight of each risk factor (*RW_i_*), that is to determine the contribution rate of different risk factors to the overall risk level, based on which the comprehensive risk level is calculated (Equation 4). According to the Borda value (*b_i_*), the judgment matrix for the risk factors is as follows:

$$B=\left[\begin{array}{ccccc} 1 & 1/2 & 1/5 & 1/6 & 1/7\\ 2 & 1 & 1/4 & 1/5 & 1/6\\ 5 & 4 & 1 & 1/2 & 1/3\\ 6 & 5 & 2 & 1 & 1/2\\ 7 & 6 & 3 & 2 & 1 \end{array}\right]$$ [Eqn 3]

Using the above judgment matrix, the weight of each risk factor can be obtained as follows:

$$RW_{i}=(0.0426,0.0629,0.1815,0.2817,0.4312)$$ [Eqn 4]

After the consistency verification, the consistency checking result of the judgment matrix is CR = 0.0265 < 0.1, indicating that the consistency test is met and that the calculation result of the influence weight of each of the above risk factors is valid.

Based on the influence weight of the above risk factors and combined with the quantised value of the risk level of each risk factor, the comprehensive risk level of the specific emergency can be obtained:

$$RL=\sum_{i=1}^{N}RL_{i}\times RW_{i}=0.7566$$ [Eqn 5]

In the specific case mentioned above, the degree of influence (*I_i_*) of the three risk factors is at ‘moderate’, one risk factor level (*RL_i_*) is at ‘relatively low’ and one risk factor level (*RL_i_*) is at ‘medium’. However, the final comprehensive risk level is above ‘relatively high’ (0.7566 > 0.7), which requires decision-makers to monitor and pay closer attention to the evolution of the event (Equation 5).

Based on the same method and procedure, the comprehensive risk level of some emergencies with the same type of the specific case was calculated. The selected cases are as follows: ‘RYB kindergarten child abuse incident’ (11/22/2017) (*RL_RYB_*), ‘Students killed in Mizhi County’ (04/27/2018) (*RL_MZ_*), ‘Girl jumped off building in Qingyang City’ (06/20/2018) (*RL_QY_*), ‘Shanghai slashing of primary school students’ (06/28/2018) (*RL*) and ‘Pupil killed in school of Shangrao’ (09/05/2019) (*RL_SR_*). Through calculation, the order of these case is *RL_MZ_ > RL > RL_SR_ > RL_QY_ > RL_RYB_*. The empirical results above show that ‘Students killed in Mizhi County’ has the highest risk level. Nine students were killed and 12 students wounded in that emergency. The high casualties cause the high-risk level. The risk level of emergencies with no casualties was lower, such as ‘RYB kindergarten child abuse incident’.

## Conclusion

In this study, we investigated 44 274 public emergencies in China over the past decade, from the perspective of managing the risks from the source and initial causes. The proposed model can help grasp the key risk factors and quantitatively assess the risk level at the early stages of emergencies, providing decision-making guidance to quickly respond to potential risks in the emergencies and to propose coping strategies according to different risk factors and risk levels, thus taking the initiative in the control of public opinion.

This research started from the ‘5W1H’ methodology. Based on the historical data of 44 274 public emergencies in China, combined with statistical analysis, correlation analysis, text analysis and expert knowledge, we have identified five major risk factors: risk time, risk location, risk population, risk psychology and risk element. Then, we build a risk matrix that is suitable for China’s national conditions based on these five risk factors. We also made a quantitative assessment of the comprehensive risk level of specific cases through a combination of the risk matrix, the Borda count and an AHP.

In the proposed model, the following highlights need to be mentioned. First, in the process of managing or controlling public opinion during an emergency, analysing historical data and summarising the experience of predecessors play a significant role. Based on the accumulated huge amount of historical data, this article identifies five types of risk factors that are then combined with expert knowledge toward the reliable establishment of risk matrix for emergencies. Second, in a traditional risk matrix, the risk level is generally divided into three levels. This article innovatively divides the risk level into five levels, namely ‘low’, ‘relatively low’, ‘medium’, ‘relatively high’ and ‘high’, which makes the assessment of risk in emergencies more accurate and more scientific. Third, through the risk matrix, the risk level of each risk factor is obtained. However, different decision-makers will focus on different risk factors, resulting in different proposals and actions. In addition, the merely mechanical summary of the risk levels for different risk factors may fail to truly reflect the overall risk level. Considering all these factors, the use of multiple quantitative methods to assess the overall risk level in this article is more scientific, objective and comprehensive. This article shows the application in public safety emergency. Further research will focus on other types of emergencies, such as the public health emergency like COVID-19. Since the global outbreak of coronavirus disease pandemic between the end of 2019 and the beginning of 2020, it has attracted extensive attention of scholars from all over the world, which is a typical major public health emergency. The World Health Organization has also announced the outbreak as a Public Health Emergency of International Concern. The ‘5W1H’ methodology and the improved 5 × 5 risk matrix will be used for risk level evaluation of the major global emergency.

The quantitative evaluation process of the risk levels in emergencies in this study is highly applicable. With its application value and practical significance, it may well improve the government’s management ability and scientific level of decision-making.
